# A Rare Case of Tirzepatide-Associated Immune Thrombocytopenia

**DOI:** 10.7759/cureus.104281

**Published:** 2026-02-26

**Authors:** Kelvin Rojas, Arshia Ahmed, Salman J Khan, Gurdeep H Singh

**Affiliations:** 1 Internal Medicine, Guthrie Lourdes Hospital, Binghamton, USA; 2 Public Health, University of Massachusetts Amherst, Amherst, USA; 3 Endocrinology, Guthrie Lourdes Hospital, Binghamton, USA

**Keywords:** diabetes treatment, drug-induced thrombocytopenia, immune-mediated side effects, mounjaro, rare side effect, tirzepatide

## Abstract

Tirzepatide, a novel dual glucagon inhibitory peptide (GIP) and glucagon-like peptide-1 (GLP-1) receptor agonist, is used in the management of type 2 diabetes mellitus (T2DM) and chronic weight management. There are no current case reports on tirzepatide causing drug-induced thrombocytopenia (DIT) despite its metabolic benefits and common gastrointestinal side effects being well documented. This case report details a 47-year-old male with a history of obesity and T2DM who presented with an ecchymotic, petechial rash within a week of increasing the dose of tirzepatide to 12.5 mg. Pertinent labs showed isolated thrombocytopenia (6000/µL), and other secondary causes of thrombocytopenia were ruled out. The patient was started on intravenous immunoglobulin (IVIG) and steroids. Platelet counts ultimately increased to baseline in response to the treatment with tirzepatide cessation, IVIG, and corticosteroids. Overall, these findings suggest an immune-mediated, drug-induced mechanism of severe thrombocytopenia. Our case intends to raise clinical awareness regarding this potential rare side effect of tirzepatide-induced thrombocytopenia. Prompt discontinuation of the drug and early initiation of steroids/IVIG can prevent life-threatening bleeding and promote recovery.

## Introduction

Diabetes mellitus (DM), especially type 2 diabetes mellitus (T2DM), and obesity are associated with significant morbidity, mortality, and healthcare costs worldwide [1]. According to the International Diabetes Federation (IDF), there were 537 million adults with diabetes globally in 2021, which is expected to rapidly increase to 783 million by 2045 [2]. Global healthcare expenditure related to diabetes was $966 billion in 2021 [2]. The obesity pandemic has further intensified this burden in the last three decades due to its role as a major risk factor for developing T2DM [1].

Similarly, diabetes is among the top causes of morbidity and mortality in the United States. The burden of diabetes has increased from 1990 to 2021 and is expected to continue to rise beyond 2045 [1,3]. In addition, the continued increase in the prevalence of obesity represents a serious threat to the national healthcare system [1]. A recent report estimated that 42.4% of adults in a surveyed group had a body mass index (BMI) consistent with obesity, which has been associated with a 3.5-4.5-fold increased risk of DM compared with people with a normal BMI [4,5].

Due to the serious burden of the epidemics of T2DM and obesity, medications with higher efficacy simultaneously in glycemic control and significant weight reduction would provide a viable option in the management of diabetic patients and have been an area of ongoing research interest. The glucagon-like peptide-1 (GLP-1) receptor agonists have already proven to be transformative in this field [6]. The glucagon inhibitory peptide (GIP)/GLP-1 dual agonist tirzepatide is a further advancement due to an additional mechanistic benefit. By activating both receptors, tirzepatide stimulates enhanced glycemic control as well as weight loss [7].

The SURPASS and SURMOUNT trials demonstrated the efficacy of tirzepatide in treating T2DM and obesity, respectively [8]. Tirzepatide was associated with better glycemic control and greater weight loss compared to placebo, semaglutide, insulin degludec, and insulin glargine [9,10]. There is also data favoring its use in cardiovascular diseases and metabolic syndrome [10]. The safety profile is generally akin to GLP-1 receptor agonist. Most adverse effects are mild-to-moderate gastrointestinal disturbances such as nausea, vomiting, and diarrhea, which are transient and dose-dependent [7].

Large-scale randomized controlled trials (RCTs) and post-marketing surveillance so far have not noted hematologic side effects, such as thrombocytopenia, in the known safety profile of tirzepatide. Real-world data analysis from the Food and Drug Administration (FDA) adverse event reporting system revealed no reported cases of thrombocytopenia or other hematologic disturbances [11]. Drug-induced immune thrombocytopenia (DIT) is a rare but potentially fatal adverse event, believed to be caused by drug-dependent antibodies. Typically, DIT causes an acute drop in platelet count and severe thrombocytopenia, with clinical features indistinguishable from immune thrombocytopenic purpura (ITP). Commonly implicated drugs include antibiotics, antiepileptics, and heparin, while tirzepatide has not previously been reported to cause DIT.

We present a rare adverse event of severe thrombocytopenia after dose escalation of tirzepatide in a middle-aged male with T2DM. A differential diagnosis of tirzepatide-induced immune thrombocytopenia was made based on the sequence of events and adequate response to steroids and intravenous immunoglobulin (IVIG). This case report is an important contribution to the current literature on the safety profile of tirzepatide and interrogates the potential need for monitoring of hematologic parameters in patients taking tirzepatide.

## Case presentation

A 47-year-old male with a medical history significant for T2DM, diagnosed three years earlier, hypertension, hyperlipidemia, anemia, obesity (BMI = 41.2), and moderate asthma/obstructive sleep apnea presented to the endocrinology clinic for the management of poorly controlled diabetes. He had no prior history of hematologic disturbances.

He had previously been treated with semaglutide, which was discontinued five months prior due to gastrointestinal side effects and dizziness. His most recent hemoglobin A1C was 12.0% (reference value: below 5.7%), and laboratory results revealed elevated microalbuminuria of 3.2 mg/dL (reference range: 0-1.9 mg/dL). He struggled with dietary noncompliance, a sedentary lifestyle, and obesity-related complications. Continuous glucose monitoring showed blood glucose levels persistently between 200 and 300 mg/dL (reference range: 70-100 mg/dL) during the preceding month.

At presentation, his diabetes regimen included metformin 1000 mg daily, dapagliflozin 10 mg daily, insulin glargine 30 units at bedtime, and oral semaglutide 3 mg daily. His other medications included lisinopril, hydrochlorothiazide, carvedilol, amlodipine, and atorvastatin. During this visit, he was initiated on tirzepatide 5 mg subcutaneously once weekly.

At his three-month follow-up, the patient reported tolerating tirzepatide 5 mg well, aside from occasional mild abdominal cramping, which he did not find concerning. The dose was subsequently titrated to 7.5 mg weekly.

One month later, a dose escalation plan was established: he was to complete 7.5 mg injections weekly for four weeks, then increase to 10 mg weekly for another four weeks, followed by 12.5 mg weekly if tolerated.

About three months later, during a follow-up for poorly controlled hypertension (home blood pressure = 180-211/107-127 mmHg), the patient’s therapy was intensified. Eplerenone 50 mg twice daily and valsartan/hydrochlorothiazide 320/25 mg daily were initiated. At this time, he was advised to increase tirzepatide to 12.5 mg weekly. 

A few days after his second dose of tirzepatide 12.5 mg, the patient contacted his primary care provider, reporting a generalized rash. He also noted a brief episode of diarrhea that had resolved spontaneously. The rash, first noticed two days earlier, had progressively worsened and now involved diffuse bruising and petechiae across his body.

Physical examination revealed widespread petechiae and ecchymoses, including on the posterior oropharynx. Laboratory testing showed a platelet count of 6000/µL (reference range: 163000-337000) (Table 1), prompting immediate referral to the emergency department (ED).

**Table 1:** Platelet trend before and during the patient’s hospital admission for thrombocytopenia.

Day of admission	Platelet count (platelets/µL) (Reference range: 163-337K)
Prior to admission	232000
Day 1	6000
Day 2	7000
Day 3	24000
Day 4	128000
Day 5	266000

In the ED, the patient denied any history of thrombocytopenia or ITP and had not experienced recent signs of infection aside from mild diarrhea. He reported no headaches, vision changes, chest pain, dyspnea, or abdominal pain. Vital signs were stable. The examination confirmed diffuse petechiae and scattered ecchymoses over the trunk and extremities.

Repeat testing demonstrated a further drop in platelets to 3,000/µL, while the remainder of his complete blood count (CBC) and cell lines were within normal limits (Table 2). CT pulmonary angiogram and CT of the head were negative for acute findings. These imaging findings effectively ruled out pulmonary embolism and intracerebral hemorrhage, which can be associated with severe thrombocytopenia. The differential diagnosis included autoimmune thrombocytopenia, idiopathic ITP, and drug-induced thrombocytopenia.

**Table 2:** Initial lab values on admission. WBC: white blood cells; eGFR: estimated glomerular filtration rate; BUN: blood urea nitrogen; AST: aspartate aminotransferase; ALT: alanine aminotransferase; INR: international normalized ratio.

Test	Results	Reference range and units
WBC count	6.94	4.23-9.07 K/μL
Hemoglobin	15.9	13.7-17.5 g/dL
Hematocrit	46.5	40.1%-51.0%
Platelet count	6	163-337 k/μL
Creatinine	0.79	0.67-1.17 mg/dL
eGFR	110	>60 mL/min
BUN	18	8-23 mg/dL
AST	22	8-33 U/L
ALT	33	10-49 U/L
Alkaline phosphatase	73	44-147 U/L
Total bilirubin	0.90	0.1-1.2 mg/dL
INR	1.07	0.86-1.11 ratio

He was treated with intravenous dexamethasone 40 mg daily, two units of platelet transfusion, and IV immunoglobulin (IVIG) under hematology guidance. He was admitted for further evaluation and management.

During hospitalization, the patient continued to receive IV dexamethasone 40 mg daily. Platelet counts gradually improved, and the rash began to resolve. Vital signs remained stable throughout admission. Hematology/oncology evaluated the patient and determined the thrombocytopenia to be secondary to tirzepatide exposure, consistent with DIT.

He completed his final dose of dexamethasone over three weeks, with platelet count recovery to 266,000/µL, and was discharged home in stable condition. He was advised to permanently discontinue tirzepatide, continue his other home medications, and follow up with hematology and his primary care provider within one week.

**Figure 1:**
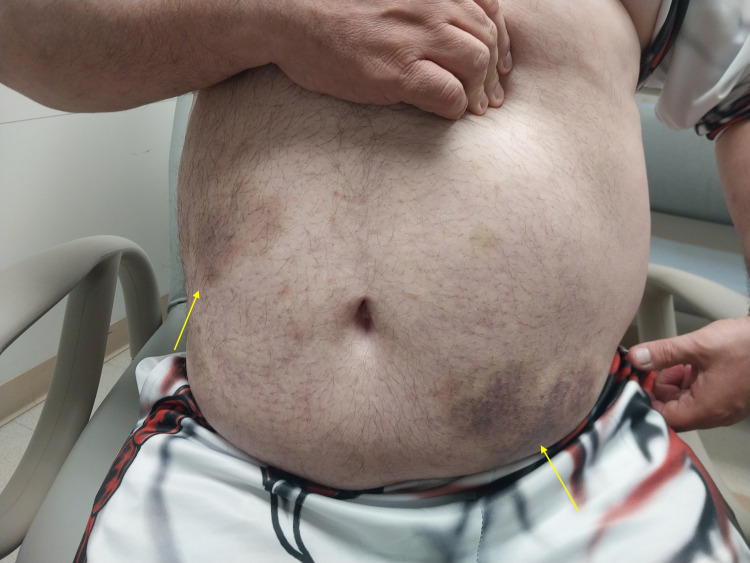
Residual ecchymosis rash (yellow arrows) over the abdomen following resolution of thrombocytopenia.

At follow-up in the endocrinology clinic one-week post-discharge, physical examination was notable only for mildly elevated blood pressure of 136/80 mmHg and residual skin discoloration along with petechial lesions (Figure 1).

The patient was counseled regarding his diagnosis of tirzepatide-induced thrombocytopenia and the need for lifelong avoidance of tirzepatide and similar agents. His diabetic regimen was adjusted accordingly, and he was instructed to continue close follow-up with hematology/oncology and primary care for ongoing monitoring.

## Discussion

DIT is a well-known cause of acute severe thrombocytopenia [12]. Several mechanisms causing DIT have been proposed, although it is not entirely elucidated [13]. One likely proposed mechanism is that the drug or one of its metabolites binds to the platelet surface proteins and induces conformational changes, causing the immune system to start making antibodies, mostly IgG and sometimes IgM. Antibodies then bind to platelets, resulting in opsonization and clearance by splenic macrophages and complement-mediated lysis [13]. Consequently, the platelet count often declines abruptly, and if levels fall below 20x109/L, bleeding manifestations are common. Halting the offending agent plays an important role in treatment, as this allows platelet recovery within days.

DIT is a diagnosis of exclusion and therefore must be differentiated from other common causes of thrombocytopenia. Immune thrombocytopenia (ITP) also presents with isolated thrombocytopenia but is not linked to a drug or medication. Heparin-induced thrombocytopenia (HIT) features a moderate drop in platelets to 40‑80 × 10⁹/L beginning five to 14 days after heparin exposure, with thrombosis often being a component [14]. Sepsis-associated thrombocytopenia is another important differential and is accompanied by fever, leukocytosis, and elevated inflammatory markers caused by a source of infection. In this case, the patient did not present with any indication of sepsis, ruling out this possibility. Disseminated intravascular coagulation is another cause of thrombocytopenia, often associated with sepsis, and leads to prolonged prothrombin time and low fibrinogen. In this condition, platelet counts improve only after sepsis is treated and resolved, which is not the case in this patient. Bone marrow failure syndromes such as aplastic anemia and myelodysplastic syndrome feature persistent thrombocytopenia and are usually accompanied by anemia or leukopenia and abnormal bone marrow morphology. In this case, the thrombocytopenia was not persistent, and the patient's hemoglobin and leukocyte count were not affected. Recognizing these differences prevents unnecessary testing and treatments and orients the clinician to the correct diagnosis. In the case presented here, other causes of thrombocytopenia were considered and ruled out, besides DIT. Nonetheless, coincidental ITP cannot be completely ruled out, although it is far less likely, given the improvement of platelet count to normal levels with discontinuation of the offending agent.

Regarding DIT, a variety of medications have been implicated. Classic agents are quinine and quinidine, which serve as the historic prototypes of immune-mediated drug-dependent platelet reactions. Other agents include antibiotics (penicillins, cephalosporins, vancomycin, trimethoprim-sulfamethoxazole, and linezolid), anticonvulsants (carbamazepine, valproate), abciximab, various chemotherapeutic agents, tirofiban, anticoagulants, and cardiovascular medications (aspirin and lovastatin) [15]. Other drugs that have rarely been reported to cause thrombocytopenia include antidepressants, thiazide diuretics, and nonsteroidal anti-inflammatory drugs [15]. DIT can occur after both initial exposure and re-exposure, and the onset is typically within five to 10 days of exposure or sooner. In this case, the patient was taking hydrochlorothiazide, which is the only other medication that has an association with DIT. However, it is unlikely that hydrochlorothiazide is the responsible offending agent since it was never discontinued, and the patient's platelet count improved despite continuing therapy. The only medication that was discontinued and whose cessation is temporally associated with the resolution of DIT is tirzepatide.

From a hematologic perspective, tirzepatide has been shown to exhibit primarily gastrointestinal side effects in major trials and has not been associated with thrombocytopenia. A literature review was done, which did not yield any similar cases except for a poster presentation of a case involving a 62-year-old male patient who also developed severe thrombocytopenia after being transitioned to tirzepatide [16]. The researchers concluded that there was level 2 evidence of DIT being linked to tirzepatide, meaning "probable" likelihood [16].

Applying the previously mentioned mechanism of DIT to tirzepatide, it is plausible that antibodies could form against tirzepatide-platelet complexes, promoting platelet destruction through Fc receptor-mediated phagocytosis or complement activation and cell lysis. Additionally, dendritic cells and T-helper cells may play a role in antigen presentation and activation of B-cell differentiation into plasma cells, thereby augmenting antibody production [17]. This is the most probable mechanism. As with other cases of DIT, the drug binds to specific epitopes on platelet surface proteins, creating new antigenic determinants recognized as foreign by the immune system, which leads to the secretion of antibodies by the immune system. Since tirzepatide is given weekly, recurrent exposure could likely give rise to and sustain this antigen-antibody interaction and contribute to platelet destruction until the drug is discontinued for a few days and eliminated.

Another potential hypothesis is tirzepatide-induced formation of immune complexes containing the drug or its metabolites and adhering to platelet surfaces, leading to complement activation and cell lysis [17]. Bone marrow suppression is unlikely, as tirzepatide has not been shown to possess myelotoxic properties in previous studies [18]. Likewise, increased platelet activation and consumption due to tirzepatide-mediated endothelial effects, or GLP-1/GIP receptor interactions leading to megakaryocytic suppression, remain speculative without supporting evidence [19]. As such, an immune-mediated antibody destruction of platelets seems the most likely mechanism for tirzepatide-induced thrombocytopenia. In this patient, no alternative cause for thrombocytopenia was identified, and the platelet decline occurred concurrently with the dose escalation of tirzepatide. The patient’s platelet count, which had dropped as low as 3000/µL, showed marked improvement following treatment with tirzepatide cessation, IVIG, and corticosteroids. This clinical course is consistent with a possible immune-mediated mechanism of tirzepatide-induced thrombocytopenia.

Suspected cases should be treated similarly to other cases of ITP or DIT. The most critical step should be the rapid identification and withdrawal of the inciting agent [9,20]. Subsequently, corticosteroids (e.g., prednisone 1 mg/kg/day) and IVIG for severe thrombocytopenia should be started. Corticosteroids are useful for suppression of the immune activity of antibodies, thereby limiting platelet destruction [20]. As for IVIG, the primary mode of action is the oversaturation of Fc receptors, which inhibits macrophage-mediated clearance of opsonized platelets [20]. However, given the extended dosing interval (once a week) and long half-life of tirzepatide, it is important to consider that platelet recovery can be delayed for several days. Once proper treatment is initiated, continued avoidance of the offending agent and platelet monitoring until recovery are paramount to avoid recurrence. This course of action was demonstrated to be effective in this patient’s case.

We present an additional case of severe thrombocytopenia related to tirzepatide dose escalation, underscoring the importance of vigilant monitoring of hematological parameters during dose titration. Target thresholds of platelet and glycemic control should be individualized to achieve the least potential for adverse events. Given tirzepatide’s widespread clinical use, attention is warranted for hematological adverse effects such as this, particularly during dose increases. Such pharmacovigilance will further elucidate its safety profile. Additional studies will be needed to clarify the risk factors and underlying mechanisms of tirzepatide-induced thrombocytopenia, hopefully leading to improved safety and monitoring recommendations in light of its growing use in clinical settings.

## Conclusions

In conclusion, this case report underscores the importance of identifying rare hematologic drug reactions in patients treated with tirzepatide. Tirzepatide generally has a favorable safety profile, but this case highlights the potential need to be cautious in using tirzepatide in patients with a known history of drug-induced thrombocytopenia. Additional studies are needed to better understand the mechanisms of tirzepatide-induced thrombocytopenia and to provide guidelines for routine hematologic monitoring. Also, important questions remain surrounding whether patients with platelet disorders should avoid agents that cause DIT and if tirzepatide should be included in this group of drugs to avoid.

## References

[1] GBD 2021 Diabetes Collaborators (2023). Global, regional, and national burden of diabetes from 1990 to 2021, with projections of prevalence to 2050: a systematic analysis for the Global Burden of Disease Study 2021. Lancet.

[2] Sun H, Saeedi P, Karuranga S (2022). IDF Diabetes Atlas: global, regional and country-level diabetes prevalence estimates for 2021 and projections for 2045. Diabetes Res Clin Pract.

[3] Huang X, Wu Y, Ni Y, Xu H, He Y (2025). Global, regional, and national burden of type 2 diabetes mellitus caused by high BMI from 1990 to 2021, and forecasts to 2045: analysis from the Global Burden of Disease Study 2021. Front Public Health.

[4] Hales CM, Carroll MD, Fryar CD, Ogden CL (2020). Prevalence of obesity and severe obesity among adults: United States, 2017-2018. NCHS Data Brief.

[5] Field AE, Coakley EH, Must A (2001). Impact of overweight on the risk of developing common chronic diseases during a 10-year period. Arch Intern Med.

[6] Nauck MA, Quast DR, Wefers J, Meier JJ (2021). GLP-1 receptor agonists in the treatment of type 2 diabetes - state-of-the-art. Mol Metab.

[7] Strollo F, Guarino G, Satta E, Gentile S (2024). Tirzepatide: a double agonist for various people living with type 2 diabetes. Diabetes Ther.

[8] Rosenstock J, Wysham C, Frías JP (2021). Efficacy and safety of a novel dual GIP and GLP-1 receptor agonist tirzepatide in patients with type 2 diabetes (SURPASS-1): a double-blind, randomised, phase 3 trial. Lancet.

[9] Del Prato S, Kahn SE, Pavo I (2021). Tirzepatide versus insulin glargine in type 2 diabetes and increased cardiovascular risk (SURPASS-4): a randomised, open-label, parallel-group, multicentre, phase 3 trial. Lancet.

[10] Ludvik B, Giorgino F, Jódar E (2021). Once-weekly tirzepatide versus once-daily insulin degludec as add-on to metformin with or without SGLT2 inhibitors in patients with type 2 diabetes (SURPASS-3): a randomised, open-label, parallel-group, phase 3 trial. Lancet.

[11] Liu L (2024). A real-world data analysis of tirzepatide in the FDA adverse event reporting system (FAERS) database. Front Pharmacol.

[12] Curtis BR (2014). Drug-induced immune thrombocytopenia: incidence, clinical features, laboratory testing, and pathogenic mechanisms. Immunohematology.

[13] Aster RH, Curtis BR, McFarland JG, Bougie DW (2009). Drug-induced immune thrombocytopenia: pathogenesis, diagnosis, and management. J Thromb Haemost.

[14] Cuker A, Arepally GM, Chong BH (2018). American Society of Hematology 2018 guidelines for management of venous thromboembolism: heparin-induced thrombocytopenia. Blood Adv.

[15] Arnold DM, Cuker A (2025). Drug-induced immune thrombocytopenia. UpToDate.

[16] Senger B, Howarth Z, Baydoun A, Rossi B (2024). Mounjaro mediated drug-induced immune thrombocytopenia. https://scholarlyworks.corewellhealth.org/cgi/viewcontent.cgi?article=1040&context=internal_medicine_posters.

[17] Thomas MK, Nikooienejad A, Bray R (2021). Dual GIP and GLP-1 receptor agonist tirzepatide improves beta-cell function and insulin sensitivity in type 2 diabetes. J Clin Endocrinol Metab.

[18] Meng Z, Yang M, Wen H, Zhou S, Xiong C, Wang Y (2023). A systematic review of the safety of tirzepatide-a new dual GLP1 and GIP agonist - is its safety profile acceptable?. Front Endocrinol (Lausanne).

[19] Mishra R, Raj R, Elshimy G (2023). Adverse events related to tirzepatide. J Endocr Soc.

[20] Bougie DW, Wilker PR, Aster RH (2006). Patients with quinine-induced immune thrombocytopenia have both "drug-dependent" and "drug-specific" antibodies. Blood.

